# External quality assurance (EQA) network in South and South-East Asia: experience and results from an international EQA programme in One Health sector reference laboratories

**DOI:** 10.1093/jac/dkaf032

**Published:** 2025-02-04

**Authors:** Tomislav Kostyanev, Hiba Al-Mir, Kristi Prifti, Lone Brink Rasmussen, Freshwork Ayalew Abegaz, Patrícia Teixeira Dos Santos, Soo-Young Kwon, Rangsiya Prathan, Taradon Luang Tongkum, Pattrarat Chanchaithong, Pitak Santanirand, Watcharaporn Kamjumphol, Ondari D Mogeni, Tobin Guarnacci, Nimesh Poudyal, Marianne Holm, Wantana Paveenkittiporn, Rungtip Chuanchuen, Rene S Hendriksen

**Affiliations:** Technical University of Denmark, National Food Institute, Kgs. Lyngby, Copenhagen, Denmark; Technical University of Denmark, National Food Institute, Kgs. Lyngby, Copenhagen, Denmark; Epidemiology, Public Health, Impact Unit (EPIC), International Vaccine Institute, Seoul, South Korea; Technical University of Denmark, National Food Institute, Kgs. Lyngby, Copenhagen, Denmark; Epidemiology, Public Health, Impact Unit (EPIC), International Vaccine Institute, Seoul, South Korea; Technical University of Denmark, National Food Institute, Kgs. Lyngby, Copenhagen, Denmark; Epidemiology, Public Health, Impact Unit (EPIC), International Vaccine Institute, Seoul, South Korea; Faculty of Veterinary Science, Research Unit for Microbial Food Safety and Antimicrobial Resistance, Chulalongkorn University, Bangkok, Thailand; Faculty of Veterinary Science, Research Unit for Microbial Food Safety and Antimicrobial Resistance, Chulalongkorn University, Bangkok, Thailand; Faculty of Veterinary Science, Research Unit for Microbial Food Safety and Antimicrobial Resistance, Chulalongkorn University, Bangkok, Thailand; Department of Medical Sciences, National Institute of Health, Bangkok, Thailand; Department of Medical Sciences, National Institute of Health, Bangkok, Thailand; Epidemiology, Public Health, Impact Unit (EPIC), International Vaccine Institute, Seoul, South Korea; Epidemiology, Public Health, Impact Unit (EPIC), International Vaccine Institute, Seoul, South Korea; Epidemiology, Public Health, Impact Unit (EPIC), International Vaccine Institute, Seoul, South Korea; Epidemiology, Public Health, Impact Unit (EPIC), International Vaccine Institute, Seoul, South Korea; Department of Medical Sciences, National Institute of Health, Bangkok, Thailand; Faculty of Veterinary Science, Research Unit for Microbial Food Safety and Antimicrobial Resistance, Chulalongkorn University, Bangkok, Thailand; Technical University of Denmark, National Food Institute, Kgs. Lyngby, Copenhagen, Denmark

## Abstract

**Objectives:**

External quality assurance (EQA) is an objective tool to assess laboratories’ diagnostic performance and their adherence to recognized international standards. External Quality Assessment in Asia (EQASIA) is an EQA network in South and South-East Asia established in 2020 with the aim of improving the quality of bacteriology diagnostics across all One Health sectors in the region. The aim of this paper is to provide a comprehensive overview of the EQA results collected from the EQASIA network and to assess improvements among the participating laboratories.

**Methods:**

Six EQA rounds were conducted between 2021 and 2023, each composed of different panels of WHO Global Antimicrobial Resistance and Use Surveillance System (GLASS) and The Food and Agriculture Organization of the United Nations (FAO) priority pathogens of interest to both the human and animal health sector.

**Results:**

Between 24 and 32 laboratories signed up for six EQA rounds (EQA1–6). Participating laboratories were able to isolate and correctly identify most of the isolates across the EQA panels except for the *Campylobacter* spp. and *Enterococcus* spp. panels. The overall performance of laboratories across the six EQAs was between 75% and 100% (average 93.3% and median 93.6%). The obtained results showed a significant improvement in laboratories’ performance over time.

**Conclusions:**

Laboratory capacity development and quality assurance in a microbiology laboratory are of particular importance especially in the context of antimicrobial resistance (AMR) and One Health surveillance. The EQASIA programme has the potential to validate laboratories’ performance in detecting important One Health pathogens, generating reliable data for effective surveillance to curb AMR.

## Introduction

External quality assurance (EQA) has a long-standing history and has played a pivotal role in laboratory medicine as an efficient and objective tool to assess laboratories’ diagnostic performance and their adherence to recognized international standards.^1–4^ There are many national and international EQA programmes across the different medical laboratory disciplines, such as clinical chemistry, haematology, pathology and microbiology, sometimes even combining some of these components into one proficiency testing (PT) round.^5,6^ In recent years, with the advancement of the molecular diagnostics and sequencing technologies, an increased need has been seen for implementing genomic PT panels, especially in genetics and microbiology.^7,8^ This is particularly important in the context of detection of antimicrobial resistance (AMR) to better assess its burden.^6,9^

To date, however, many laboratories, mainly in low- and middle-income country (LMIC) settings, rely solely on phenotypic methods for the detection of microbial pathogens. Therefore, PT schemes that target the phenotypic aspects of microbiological diagnostics such as identification of the species, sub-typing and antimicrobial susceptibility testing (AST), remain currently in the core of EQA programmes in microbiology. Such a programme was established in 2020 with support from the UK Aid’s Fleming Fund (FF)^10,11^ and with the overall aim to improve the quality of bacteriology diagnostics through implementing EQA provision across all One Health (OH) sectors in South and South-East Asia.^10^ The FF grant was awarded to the Strengthening External Quality Assessment in Asia (EQASIA) project and EQASIA is currently supported until the end of 2025.^10^ In its initial phase, EQASIA conducted an in-depth mapping of available EQA services across the South and South-East Asian region. National reference laboratories (NRLs) and centres of excellence (CoE) were identified and gradually included in the EQASIA network for the participation in the EQA programme.^10^ The main goal of the project was to provide EQA panels on a regular basis to both human health and animal health microbiology laboratories in the region but also to strengthen existing national EQA activities. In addition, capacity building and training activities were in the scope of EQASIA as part of a tailored follow-up approach based on laboratories’ performance in each EQA round.^12^

The aim of this paper is to provide a comprehensive overview of the EQA results collected from the EQASIA network during six EQA rounds from 2021 until 2023 with the specific objective to assess improvements among the participating laboratories. The article also describes in detail the structure and design of the EQA programme including an elaboration on how EQASIA addressed underperformance in the participating laboratories. Finally, the paper underlines the importance of such an EQA programme in South and South-East Asia and addresses plans for it to become sustainable.

## Materials and methods

### EQA panel design and preparation

**Figure 1.**
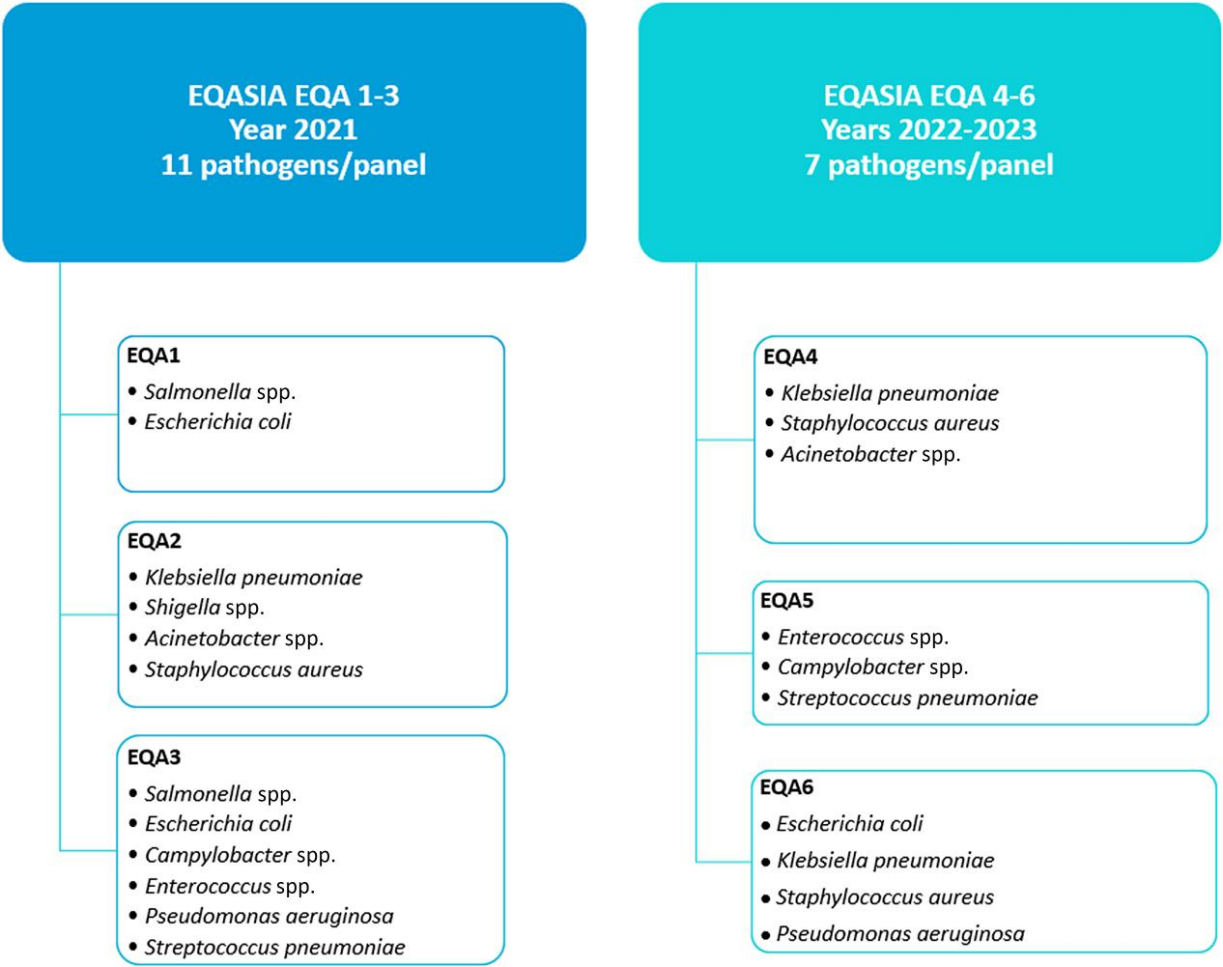
EQASIA EQA rounds (EQA1–6) and their composition.

Since the inception of the EQASIA programme in 2020, six rounds of EQAs (EQA1 to EQA6) have been prepared and conducted targeting human and animal health as well as food safety and environmental laboratories in the South and South-East Asian region. Each round was composed of different panels of WHO Global Antimicrobial Resistance and Use Surveillance System (GLASS) and The Food and Agriculture Organization of the United Nations (FAO) priority pathogens of interest to both the human and animal health sector (Figure 1). Initially, for EQA1–3, each panel (except for the matrix panel) included 11 bacterial strains of which only eight strains were the targeted species (i.e. *E. coli*). The other three strains were non-target species (e.g. *Klebsiella*, *Salmonella*, etc. for the *E. coli* target). Subsequently, for EQA4–6, the number of target strains in each panel was reduced to five and the number of non-target strains to two to limit the workload for participating laboratories. The target species for each panel represents a heterogeneous selection with different phenotypic profiles and underlying resistance mechanisms. One of the target strains in each panel was used as an internal control and was included every time the respective panel was part of an EQA round (i.e. *E. coli* strain X in EQA1, EQA3 and EQA6). A list of provided isolates and their susceptibility profile is available in the Supplementary data (available as Supplementary Table S1 in the Supplementary data at *JAC* Online).

Candidate strains for each EQA were selected among the existing collection of strains at the Technical University of Denmark, National Food Institute (DTU Food) or procured from partner institutions after signing a Material Transfer Agreement. The isolates were from human or animal origin and were well characterized by using MICtesting and WGS. They were tested at DTU and additionally verified at The Peter Doherty Institute for Infection and Immunity, Melbourne, Australia. Expected MIC values were identified for a range of antimicrobials relevant for the different species (Supplementary data). Subsequently, the identification and AST profile of the selected strains for each panel were further confirmed by the Faculty of Veterinary Science, Chulalongkorn University, Bangkok, Thailand (CUVET) and the National Institute of Health, Bangkok, Thailand (NIH). A consensus of the results was sought between the different partners, which helped with choosing the desired final number of isolates for each panel (i.e. 11 for EQA1–3 or seven for EQA4–6). The final set of results was designated as expected or baseline results that the results from the participating laboratories were compared to. The obtained AST results were interpreted based on most up-to-date breakpoint values developed by the CLSI (CLSI M100).^13^ When not available, EUCAST clinical breakpoints^14^ or epidemiological cut-off values (https://mic.eucast.org/) were applied instead.

The EQA programme was designed as a ‘One-Shop EQA’ where participating laboratories could choose to receive one or more of the available panels when signing up for an EQASIA EQA round using the DTU informatic EQA system. The EQA isolates were lyophilized by NIH and CUVET and sent to participating laboratories from the human health and animal health sector, respectively. Participants were provided with an online available protocol containing clear instructions in English how to revive and process the strains using their routine procedures for identification and AST, and how to submit the results to the online EQASIA Informatics Module. Subject to AST were only the target strains (i.e. eight out of 11 isolates in each panel for EQA1–3 and five out of seven pathogens for EQA4–6). Participants were encouraged to test as many of the antimicrobials listed as possible considering their relevance regarding the laboratory’s routine work. Reference strains of the ATCC, relevant to each of the EQA panels sent, were provided to all participants free of charge with instructions for storage and maintenance for quality assurance purposes and for future EQA trials.

### Data collection and verification

Participating laboratories were instructed to submit the EQA data into an online tool, the EQASIA Informatics Module developed at DTU Food in the frame of the project. The tool was designed to collect information on methods used when processing the EQA strains locally, as well as results on identification, AST and reference strain testing. In addition, other EQA panel-specific information was captured, i.e. beta-lactamase (ESBL/AmpC/carbapenemase) production for Enterobacterales isolates, serotyping for *Salmonella* spp., etc. (data not included). After the submission deadline, results were reviewed and in case of missing or incomplete data, laboratories were queried or asked to (re-)submit the results before result evaluation and publication of expected results and individual scores.

### Evaluation of results and data analysis

Results were automatically evaluated by the online tool after comparing them to the expected baseline results. In EQA1–2, results in agreement with the expected interpretation were categorized as ‘1’ (correct), while results deviating from the expected interpretation were categorized as ‘0’ (incorrect). In the subsequent EQA3–6, a more discriminatory approach for evaluation was used for the AST results. Categorical agreement and rates of minor, major and very major errors were attributed according to ISO 20776–2:2007. Thus, results in agreement with the expected interpretation were categorized as ‘4’ (correct), while results deviating from the expected interpretation were given a score of ‘3’ (incorrect, minor error), ‘1’ (incorrect, major error) or ‘0’ (incorrect, very major error) (Table 1). The sum of all evaluated AST results generated an overall performance score, based on which laboratories could be compared and ranked. All participating laboratories were issued a certificate showing the number of AST tests performed and the overall performance rate for each EQA panel, separately.

**Table 1.** Distribution of scores according to the reported results and their agreement with the baseline expected results

Scores		Obtained interpretation		
		Susceptible	Intermediate	Resistant
Expected Interpretation	Susceptible	4	3	1
	Intermediate	3	4	3
	Resistant	0	3	4

The overall data were collated, cleaned and analysed using R Statistical Software version 4.2.3.^15^ The overall performance of laboratories was calculated based on their AST score for each and throughout all EQA panels. A heatmap was generated to visualize laboratories’ performance in each of the pathogens included in EQA1–6. To determine whether the observed differences in performance at each time point were statistically significant, the Friedman test was used as the data did not meet normality assumptions. The laboratories’ performance when testing certain EQA pathogens (i.e. *E. coli*, *K. pneumoniae*, *Salmonella*, *S. aureus*) was compared across the different panels. The assessment also looked in depth into the internal controls included in each panel for a comparison of the laboratory performance on these specific isolates.

## Results

### EQA participation

**Figure 2.**
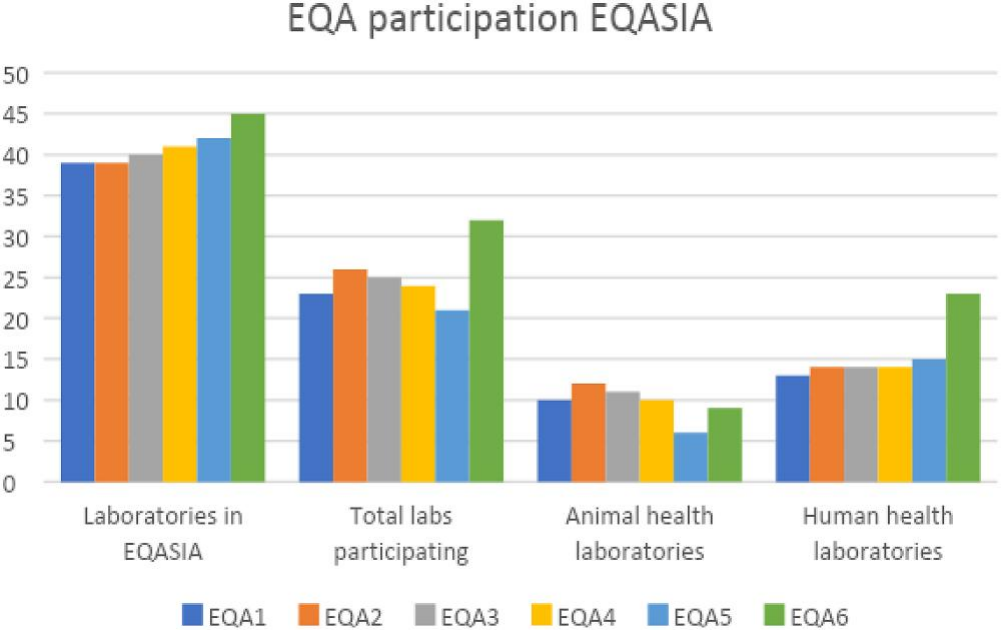
Distribution of participating laboratories (*n*) across the six EQASIA EQAs. Food safety microbiology laboratories and environmental laboratories were included within the AH group for the data analysis.

The EQASIA network started with 39 laboratories from the South and South-East Asian region in 2021 and expanded to include 45 laboratories in 2023. The list of countries and number of laboratories per EQA round is presented in the Supplementary data. The number of laboratories that signed up for the different EQA rounds (EQA1–6) varied between 24 and 32 (Figure 2 and Figure S1).

**Figure 3.**
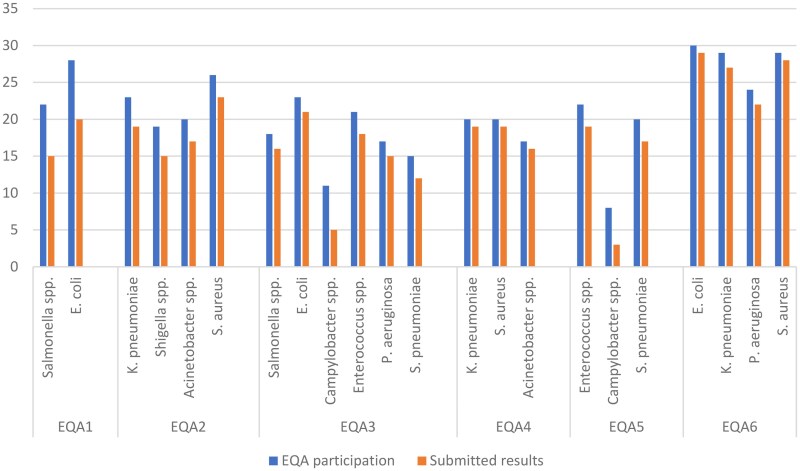
Number of laboratories that signed up per EQA panel (EQA participation) and submitted their results.

The participation rate was between 57% (EQA5) and 74% (EQA1) of the laboratories included in the EQASIA programme at the time of conducting the PT. Between 79% of participating laboratories in EQA1 and 97% in the latest EQA6 submitted results (Figure 3).

### Identification of EQA strains

**Figure 4.**
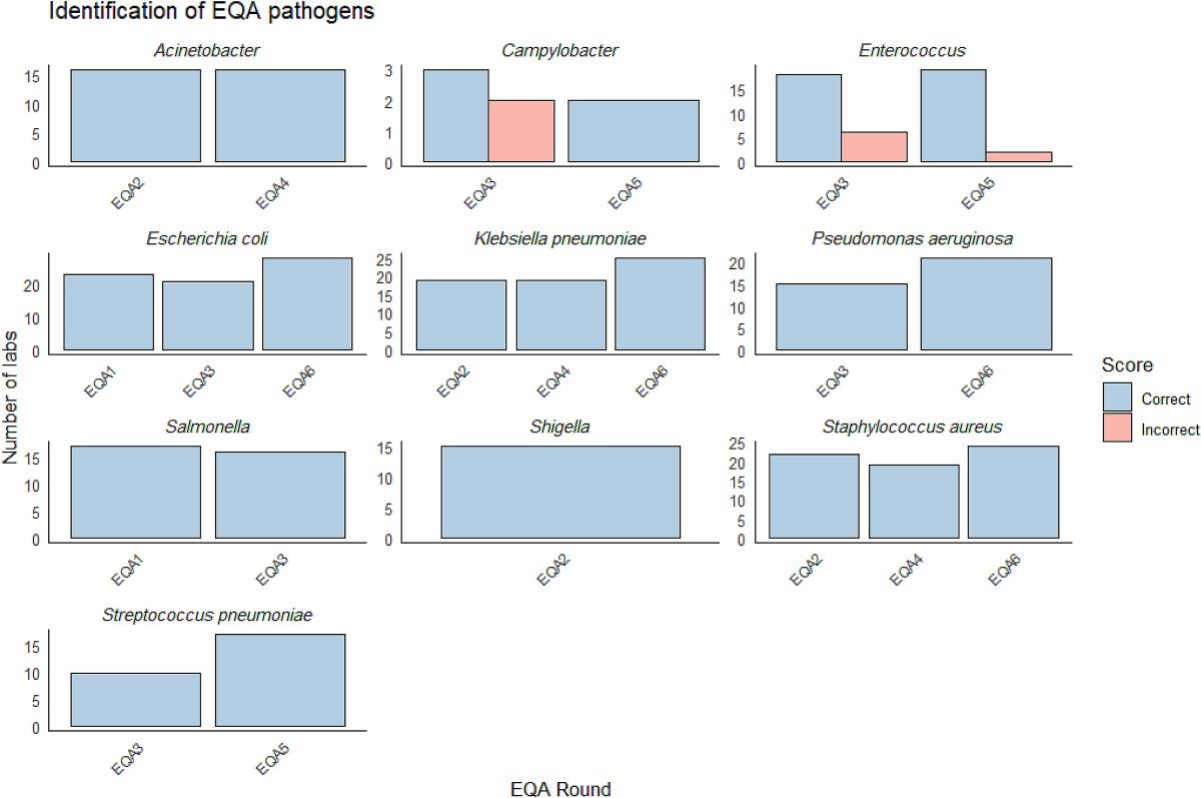
Laboratories’ performance in the identification component in EQA1 to EQA6 presented by pathogen.

Overall, participating laboratories were able to isolate and correctly identify most of the strains across the different EQA panels except for the *Campylobacter* spp. and *Enterococcus* spp. panels (Figure 4).

### Antimicrobial susceptibility testing

**Figure 5.**
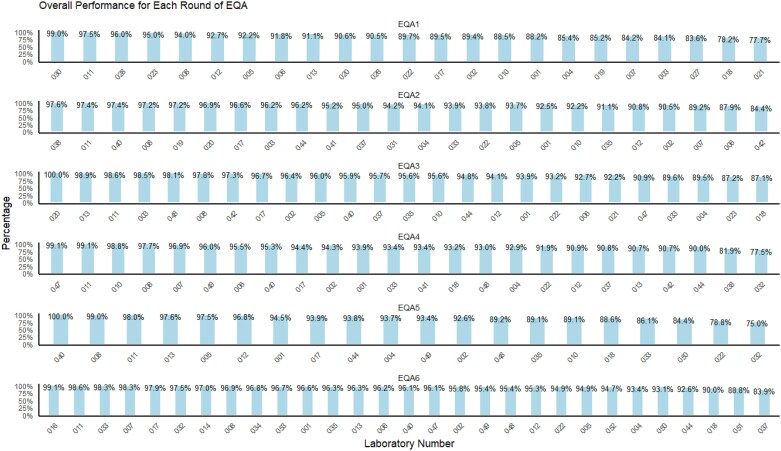
Performance of all participating laboratories in the different EQASIA EQAs.

The overall performance of laboratories across the six EQAs was between 75% and 100% (average 93.3% and median 93.6%). Broken down by EQA, it varied between 83.6% and 99.0% (EQA1), 84.4% and 97.6% (EQA2), 87.2% and 100% (EQA3), 77.5% and 99.1% (EQA4), 75.0% and 100% (EQA5), and 83.9% and 99.1% (EQA6) (Figure 5).

Participants demonstrated the highest performance when testing the *E. coli* panels (performance ranged from 90.4% and 100%) and the lowest performance in the *Campylobacter* spp. panel (average 74.3%). The overall performance of laboratories per EQA and pathogen is shown in the Supplementary data (Figure S2). Some of the lowest performance scores (light blue/grey) were in the *Campylobacter* spp. panel for laboratories #033 (50.0%) and #006 (66.7%), *Salmonella* spp. panel for laboratory #007 (60.0%), *Enterococcus* spp. panel for laboratory #032 (65.6%), and *S. pneumoniae* panel for laboratory #022 (66.4%). Based on these scores, up to four underperforming laboratories were identified in each EQA round. These laboratories were subject to additional training and follow up provided by EQASIA.

There were eight laboratories (EQASIA #001, #002, #004, #008, #011, #012, #017 and #022) that participated throughout all six EQA rounds. They had selected different pathogens to be tested across these EQAs. Their overall performance for each EQA was calculated and compared (Supplementary Figure S3). EQA1 displayed the widest interquartile range (IQR) of scores, indicating more variability in laboratory AST performance among laboratories. From EQA2 to EQA6, the performance seemed to stabilize with narrower IQRs, suggesting improved consistency over time. In EQA5 and EQA6, the boxplots exhibited higher median scores, indicating an improvement in overall laboratory AST performance scores. Laboratory EQASIA #022 had the lowest score in EQA5 (78.8%). The highest score was 99.1% of laboratory EQASIA #011 in EQA4.

The Friedman test showed statistically significant differences of performance across the six EQA rounds (Friedman chi squared = 15.3, *P* value = 0.009209). To look more in depth between which times points the most differences were, *post hoc* analysis was conducted as pairwise comparisons using Conover's all-pairs test for a two-way balanced complete block design. The most significant differences were between EQA1 and EQA3 with *P* = 0.03 and EQA1 and EQA6 with *P* = 0.0002. In addition, to account for random variation across different laboratories, a mixed-effect model was used. This model also suggested that, on average, there was a significant improvement in performance across time points compared to EQA1, with varying degrees of change. However, the presence of random effects for laboratories indicates that there is significant variability between laboratories, which is important to consider when interpreting the overall performance changes. The estimated effects of each EQA on the performance relative to EQA1 are shown in Table 2. This model is effective in parsing out the time effects from the variability due to different laboratories, providing a clearer picture of how performance metrics change over time independent of the laboratory variations.

**Table 2.** Estimated effects of each EQA on the performance in relation to EQA1 in EQASIA

EQA	Estimated effect	*P* value	Interpretation
EQA2	3.308	0.015	A significant increase indicating that scores at EQA2 are on average 3.308% higher than at EQA1
EQA3	4.204	0.0026	More substantial and significant increase
EQA4	3.569	0.0093	Significant increase
EQA5	2.623	0.0515	Suggestive increase, approaching significance
EQA6	5.363	<0.0002	Largest and highly significant increase

**Figure 6.**
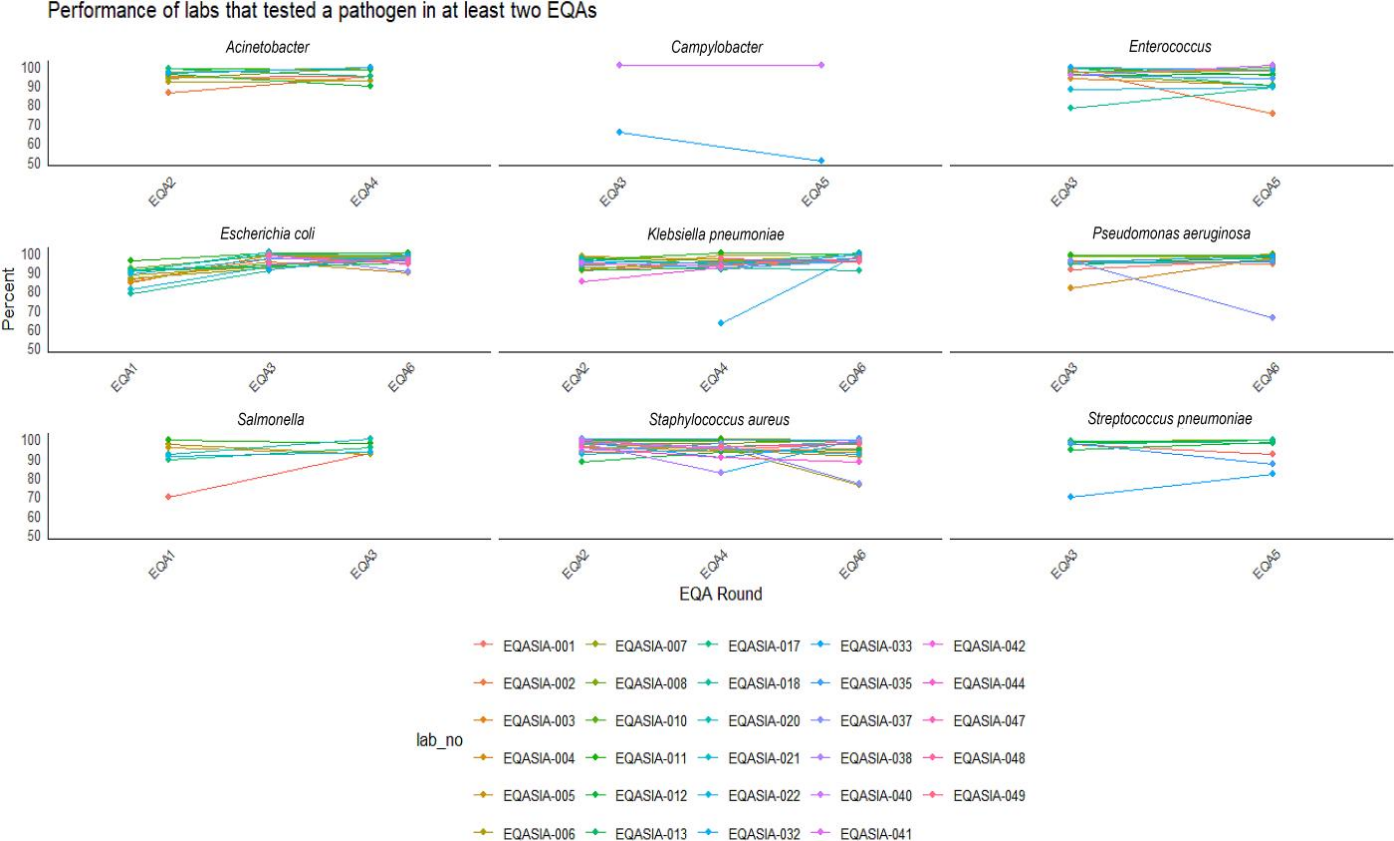
Performance of laboratories (*n *= 29) that tested any given pathogen in at least two EQAs.

For the following part of the analysis, only data from at least two timepoints were included, where available (Figure 6). Most of the laboratories that participated in all EQA rounds showed the same or improved performance with time. There were, however, a few exceptions, i.e. laboratories ## 002 (performance decreasing from 97.6% to 75.0%) and 017 (from 99.0% to 89.0%) for the *Enterococcus* spp. panel and laboratory #012 (from 95.6% to 89.1%) for the *Acinetobacter* spp. panel. Laboratories’ performance on individual EQA pathogens was done by comparing their AST score from at least two different timepoints (i.e. *Acinetobacter* spp. in EQA2 and 4).

**Figure 7.**
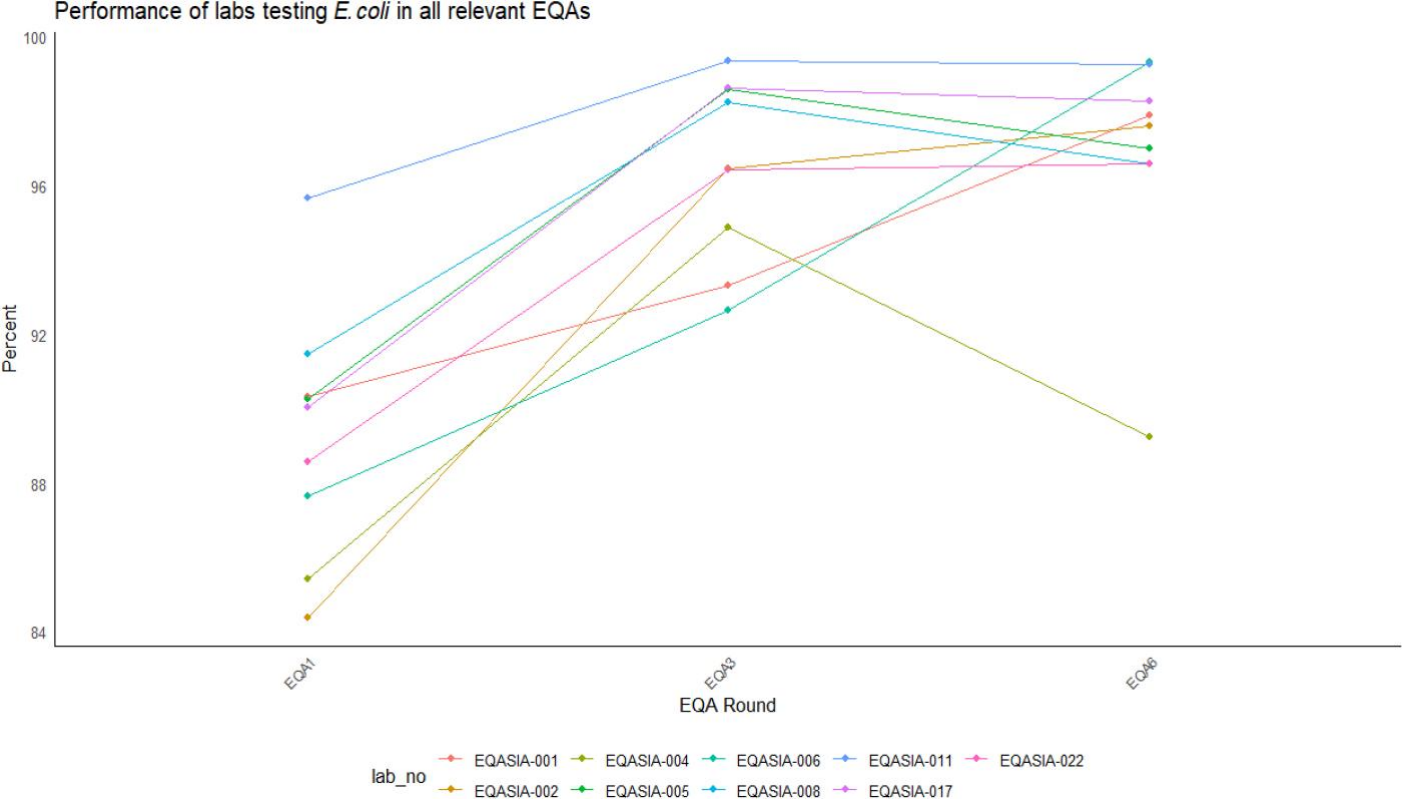
Performance of laboratories (*n* = 9) across the *E. coli* EQA panels. Only laboratories that reported data from three time points were included.

For the *E. coli* panel, there were nine laboratories that participated in all three EQA timepoints. There were significant differences of AST performance scores between EQAs for *E. coli* as shown by the Friedman test [chi squared (2) = 13.556 and *P* = 0.001139]. The *post hoc* analysis showed that the significant improvement is between EQA1 and EQA3 (+7.19%), and between EQA1 and EQA6 (+7.55%). (Figure 7).

**Figure 8.**
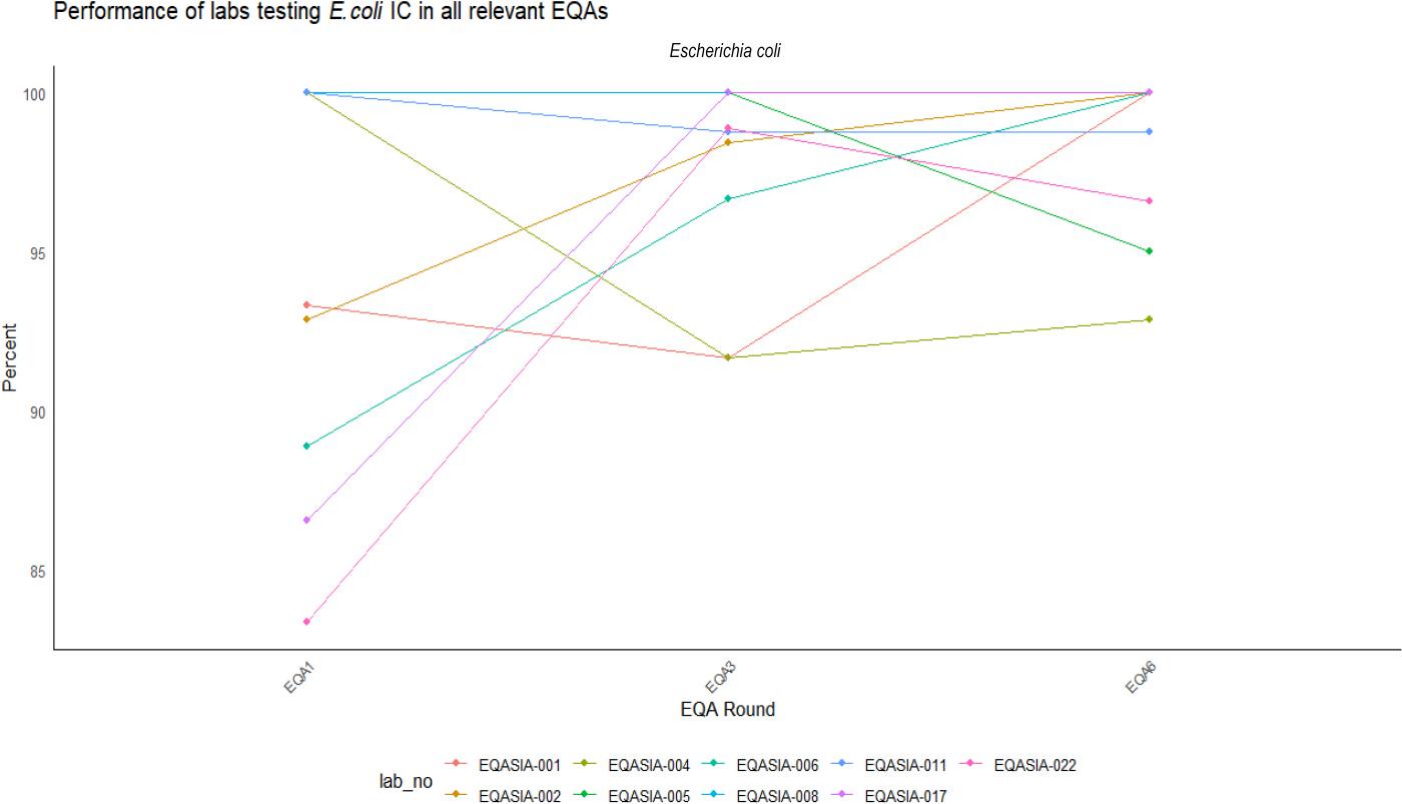
Performance of laboratories (*n* = 9) testing *E. coli* panels in EQA1, EQA3 and EQA6. Performance rate on the testing of the IC is presented.

Each EQA panel included an isolate used as an internal control (IC) that was added every time the respective pathogen was part of an EQA round. There were nine laboratories that consistently participated in all three *E. coli* EQAs and their performance on the IC strain showed no statistically significant differences between EQAs (Friedman chi squared = 0.89655, degrees of freedom = 2, *P* = 0.6387) (Figure 8).

## Discussion

Laboratory capacity development and quality assurance in a microbiology laboratory are of particular importance especially in the context of AMR and OH surveillance.^16^ EQA programmes play an important role in ensuring certain standards of quality are met by the participating laboratories, highlighting potential pitfalls and gaps in their diagnostics, as well as improving laboratories’ performance in detecting certain pathogens and resistance profiles.^2–4^ Participation in international EQA networks provides opportunities and is beneficial for laboratories especially from LMIC settings.^10,17,18^ Importantly, providing PT panels along with quality control strains to participants free of charge and placing an emphasis on continuous capacity building would ultimately lead to improved diagnostics of AMR and shortening the gap between the Global North and the Global South.^19,20^

The EQASIA project has conducted six EQAs that covered up to 32 laboratories located in 14 countries in South and South-East Asia. Large differences were observed in their performance throughout these EQA exercises mainly due to the differences in their background, i.e. processing samples from either the HH or AH sector, but also because of the different levels of implementation of their quality management system.^12^ Most laboratories were either NRLs or CoE, some of which were also national EQA (NEQA) providers that were running EQA schemes for a network of laboratories in their country. To increase its coverage, EQASIA has also included several sentinel laboratories in need of capacity building and support that participated in EQA6 and subsequent EQAs (data not included in this study). Overall, these laboratories were less prepared to engage in an EQA PT and their score was lower than the one of the laboratories with longer participation in the EQA programme. This underlines the importance that these laboratories should have access to and participate in EQA programmes, which would improve their laboratory performance, monitor their competence and enhance the quality of their diagnostic services.^21^

On the one hand, the EQASIA programme offered participating laboratories a flexible scheme enabling them to choose one or more panels among several options in each EQA round. This allowed for a more tailored approach according to the profile of each laboratory. On the other hand, this could have actually hidden potential weaknesses in laboratories’ capacity to detect certain pathogens and resistant types. Overall, no major issues were observed in the identification component of the six EQA rounds. Most of the pathogens included in EQASIA were relatively straightforward and easy to identify (i.e. *E. coli, S. aureus*, etc.) and did not pose problems to the participating laboratories. However, the success rate in the identification of the *Enterococcus* spp. and *Campylobacter* spp. panel was lower because the laboratories had to distinguish between two target species included in these EQA panels (*E. faecalis* and *E. faecium*, and *C. coli* and *C. jejuni*, respectively). These panels revealed several quality issues in some laboratories that were tackled and addressed in the feedback given to them. Laboratories’ performance in the AST component of the EQA rounds varied according to the composition of the proficiency panel chosen. The average success rate was the lowest in the *Campylobacter* spp. panel (74.3%), followed by *S. pneumoniae* and *Enterococcus* spp. However, the *Campylobacter* spp. panel was sent out in only one of the six EQA panels and was chosen by only six participants. The challenges that laboratories had in identifying and performing susceptibility testing of *Campylobacter* spp. isolates could be a potential pitfall when participating in international surveillance programmes, i.e. WHO GLASS and FAO InFARM. Overall, we could observe that there was a significant improvement in laboratories’ performance across the time points compared to EQA1, with varying degrees of change. The significant results for the EQAs suggest that any interventions or conditions represented by these different rounds have measurable effects on the outcomes.

EQASIA has provided participating laboratories with ATCC reference strains for quality control testing. These isolates were sent along within each EQA round to incentivize laboratories to conduct QC regularly while performing AST. Most of the participating laboratories reported results; however, a handful of laboratories did not perform any testing of ATCC reference strains mainly due to inability to store and re-use them in subsequent EQAs and in their routine practice. Of the reported results, it should be noted that in some laboratories the panel of antibiotics that was subject to quality control, and hence reported within the EQA round, so they did not necessarily match with the panel of antibiotics tested against the EQA strains. There were also several laboratories that reported results outside of the acceptance range for some antibiotics. For the laboratories using automated methods determining minimum inhibitory concentration, this could have been due to software not up to date and in line with the latest CLSI or EUCAST ranges, as well as other reasons, i.e. density of the inoculum. For the laboratories, using disc diffusion methodology, this could have occurred because of various reasons for troubleshooting when performing AST. To the best of our knowledge, the laboratories reporting results out of range had not undertaken immediate corrective and preventive actions at the point of testing to identify the reasons for the deviations in these results.^12^

A threshold of 95% performance score (5% deviation in the AST results compared to the baseline results) was set across each of the six EQA rounds to identify laboratories that need attention and capacity building to further improve their performance in EQAs and in general.^22^ Three to four laboratories from the HH sector and one to two laboratories from the AH sector with the lowest performance score were given individualized feedback on their performance after each EQA round and subsequently online consultation meetings were conducted with them. The overall aim was to highlight gaps in their AST procedure that could be addressed by each laboratory. An added value of EQA schemes is the ability to flag potential underperforming laboratories that could be submitting surveillance data of lower quality, which can hamper local infection prevention and control but also other interventions and efforts to mitigate AMR spread locally. In addition, other types of training activity (i.e. educational webinars, workshops and refresher training) were offered by the EQASIA project that complemented the EQA programme.

Overall, there was a substantial diversity in performance and proficiency among participating laboratories. During the EQA programme, we could observe an improvement in the performance score in all laboratories that had a deviation of >5% from the baseline results. This underlines again the importance for an EQA programme to provide appropriate feedback, follow up and capacity building while supporting laboratories in their efforts to improve the overall quality of diagnostics. We also observed a significant improvement of performance of laboratories that participated systematically in all EQA rounds of the programme. This supports the added value of such an EQA scheme that needs to become sustainable and provide EQA pathogens to laboratories in the region on a regular basis. If extended beyond its current horizon, EQASIA has a potential to complement and even to replace other minor and insufficient EQAs running in the region. EQASIA could also benefit major players in the AMR field by validating microbiology laboratories’ performance when it comes to identification and AST of important OH pathogens. This is especially important for NRLs that systematically report surveillance data to GLASS, InFARM and other data collection initiatives. Ultimately, this would allow laboratories to generate reliable data that can confidently be used for effective surveillance to curb AMR.

## Supplementary Material

dkaf032_Supplementary_Data

## Supplementary data

Figures S1–S3 and Table S1 are available as Supplementary data at *JAC* Online.
