# Supplementary material for: External quality assurance (EQA) network in South and South-East Asia: experience and results from an international EQA programme in One Health sector reference laboratories
Source: J Antimicrob Chemother. 2025 Feb 4;80(4):1037–46. doi: 10.1093/jac/dkaf032 (PMC11962387; doi:10.1093/jac/dkaf032)
Supplement: dkaf032_Supplementary_Data [file dkaf032_supplementary_data.docx]

**Supplementary data**

**Supplementary table.** MIC values and interpretation of the selected strains included in each EQA round.

**EQASIA EQA1**


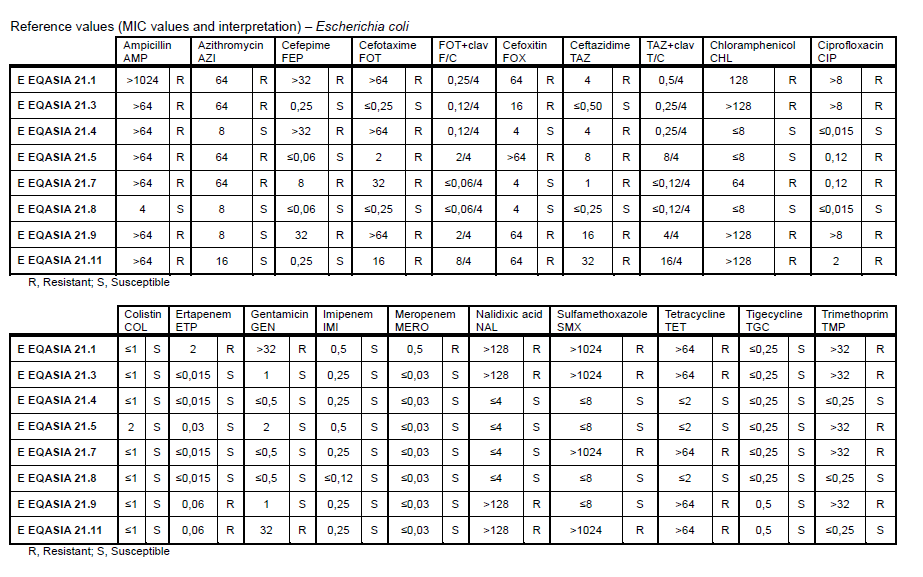


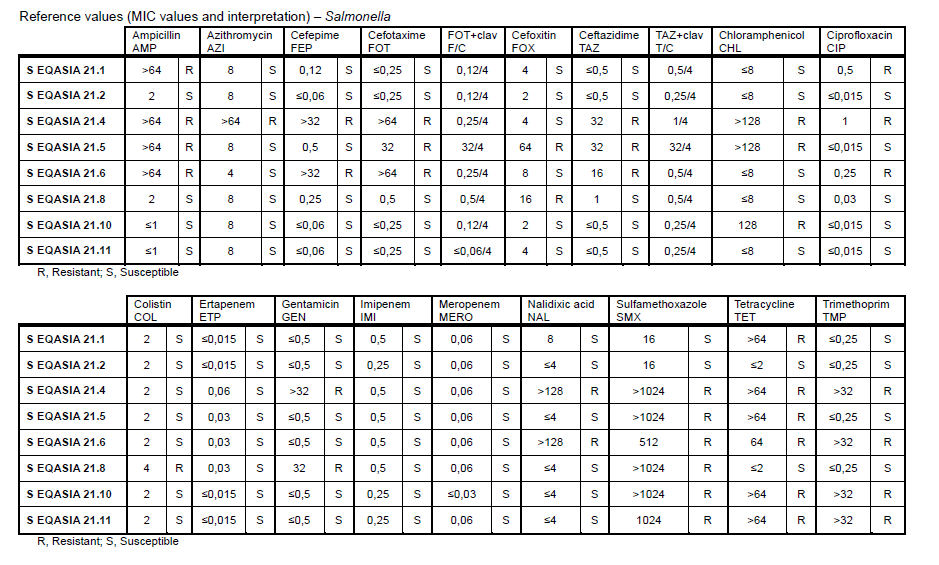


**EQASIA EQA2**

**
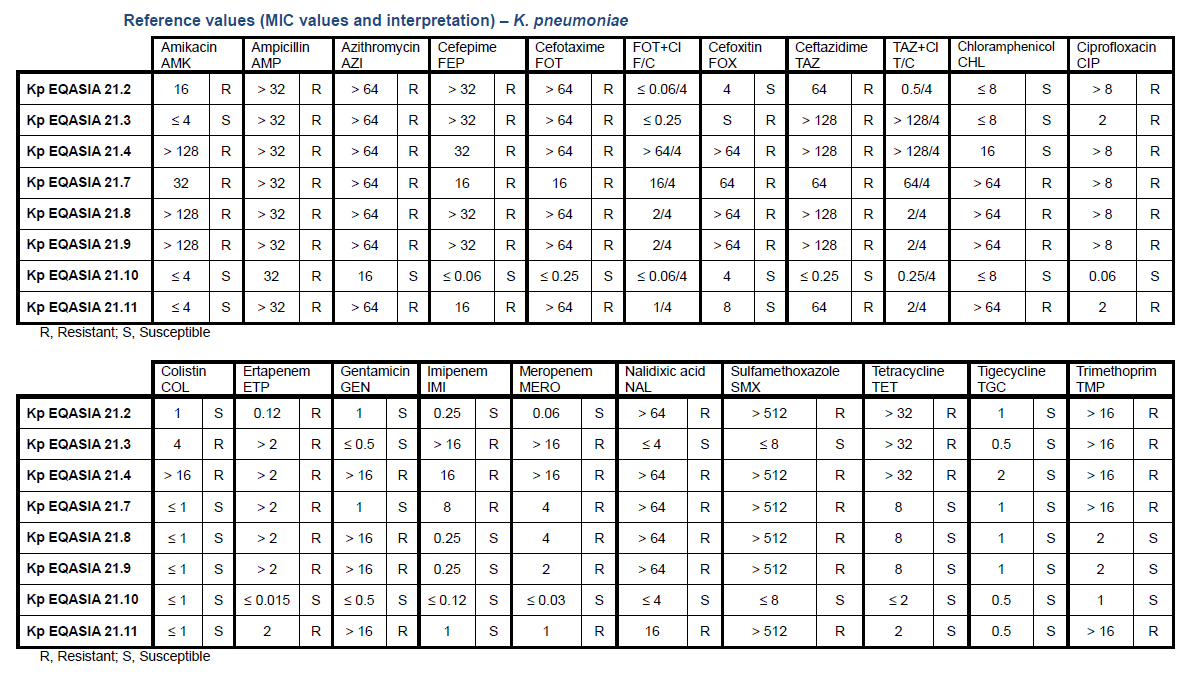
**

**
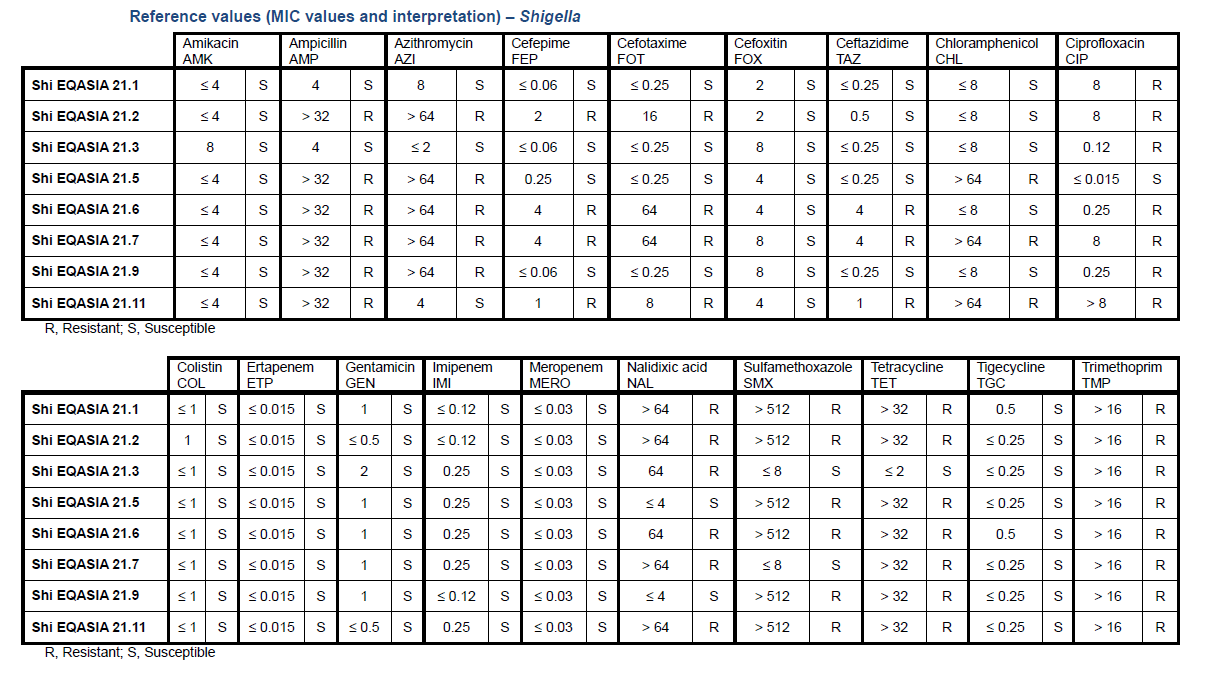
**

**
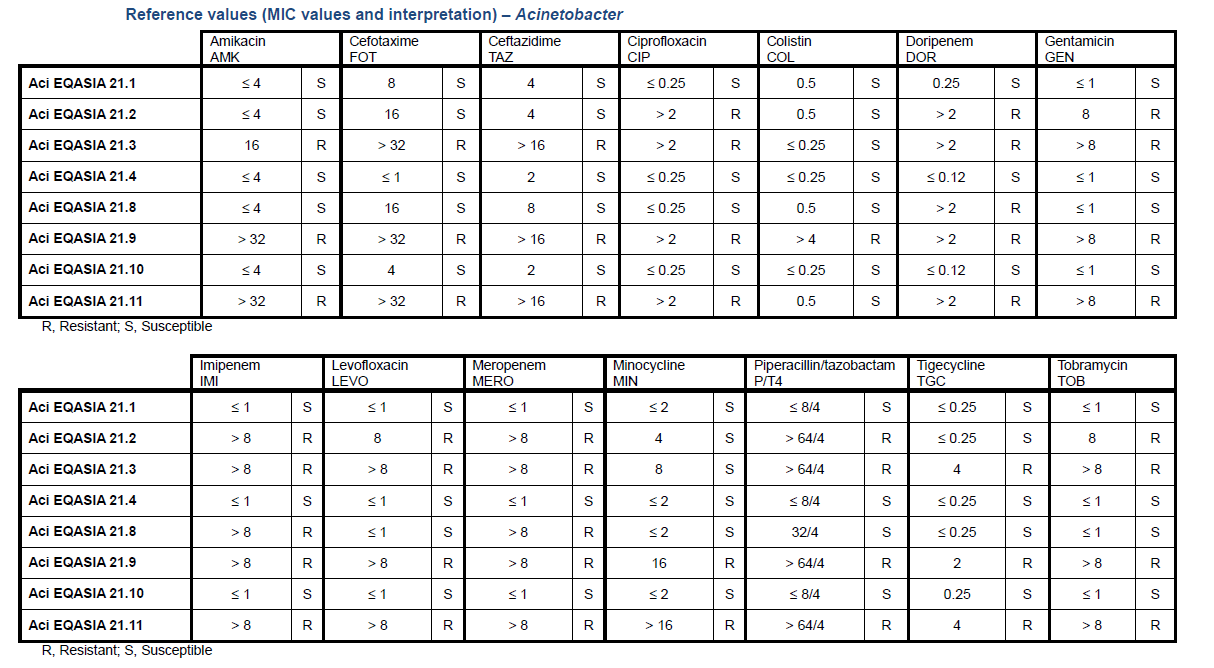
**

**
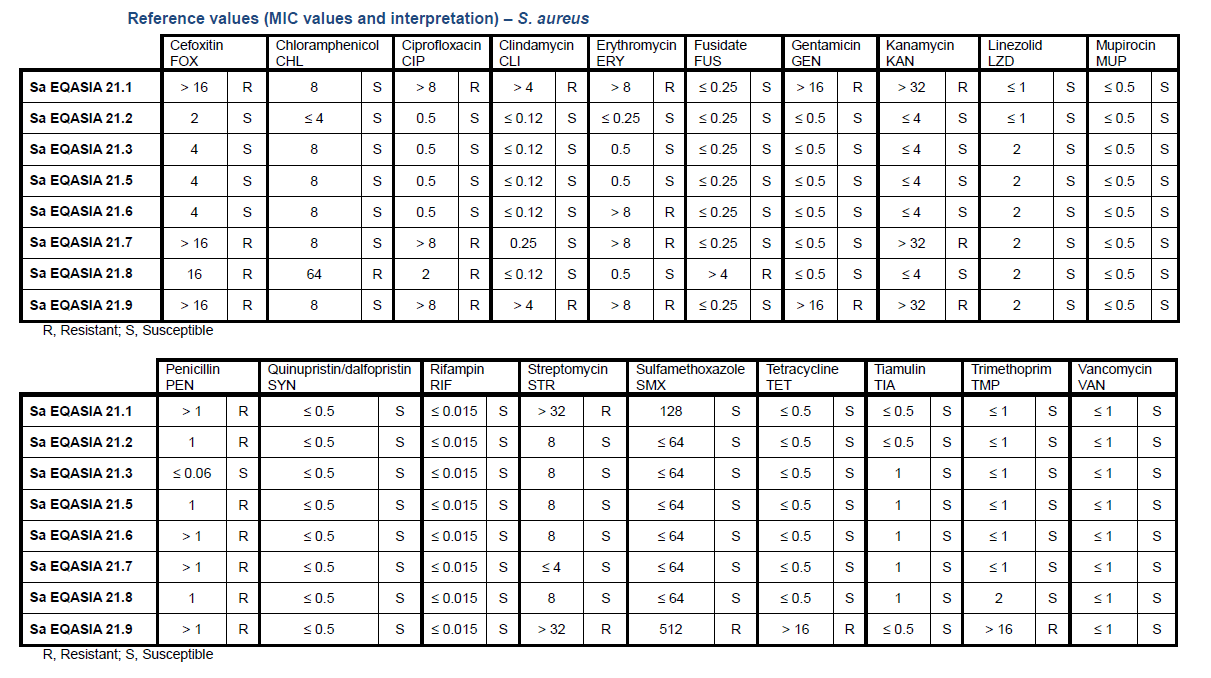
**

**EQASIA EQA3**


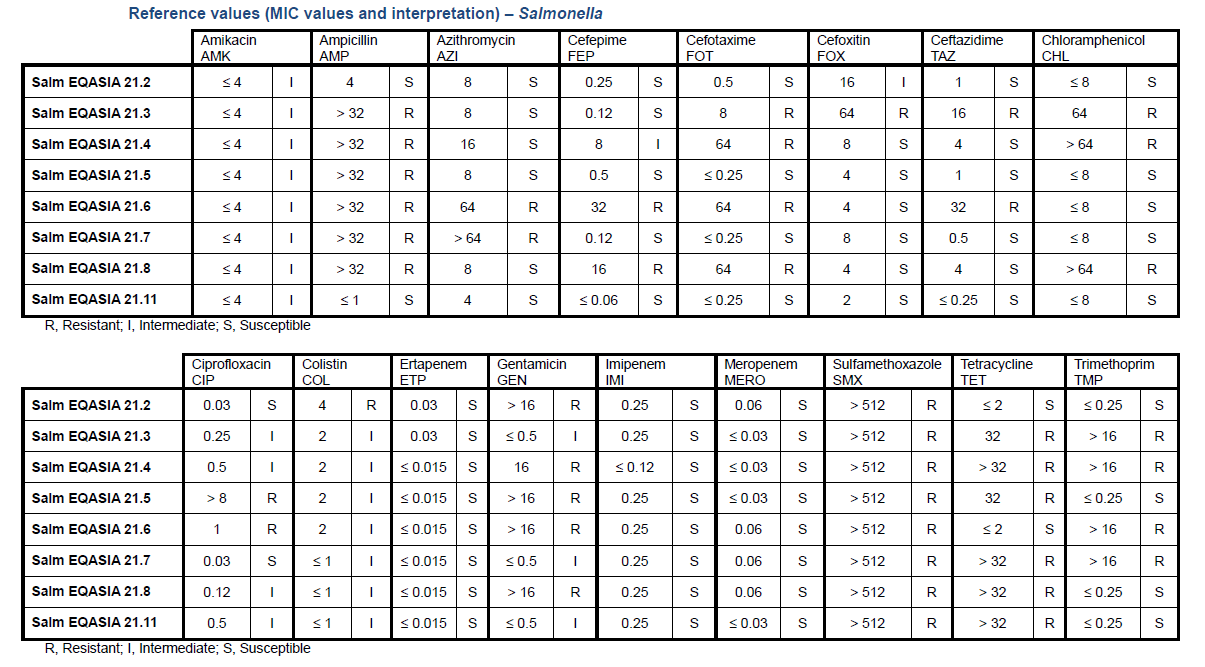


**
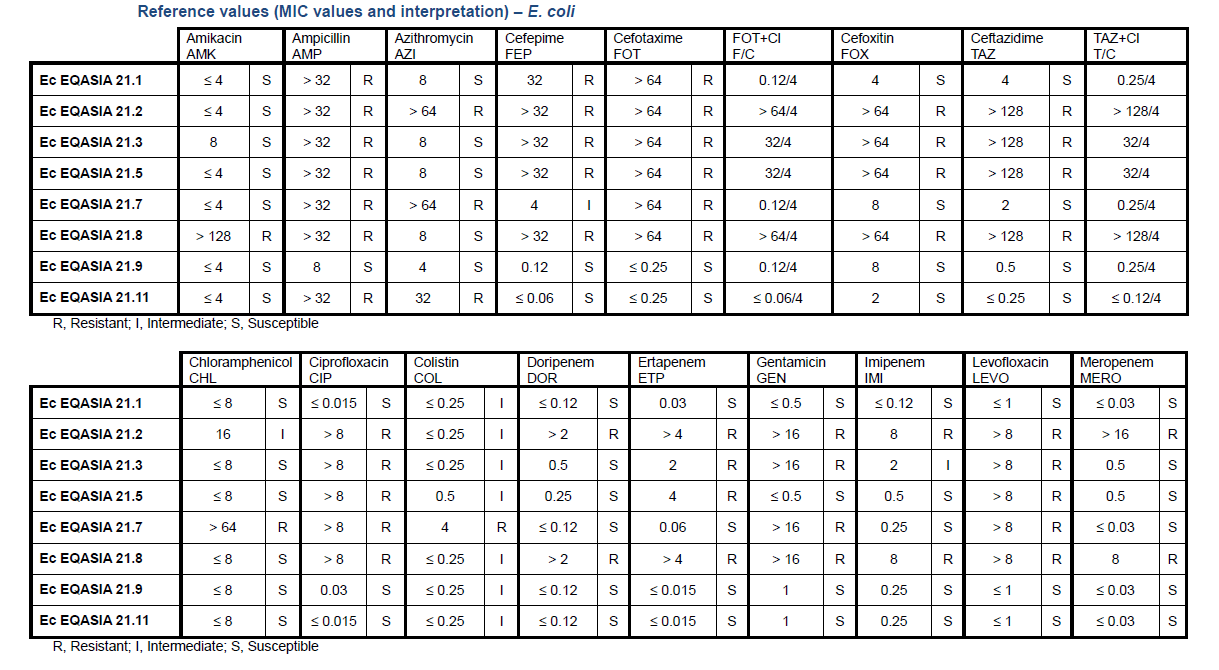
**

**
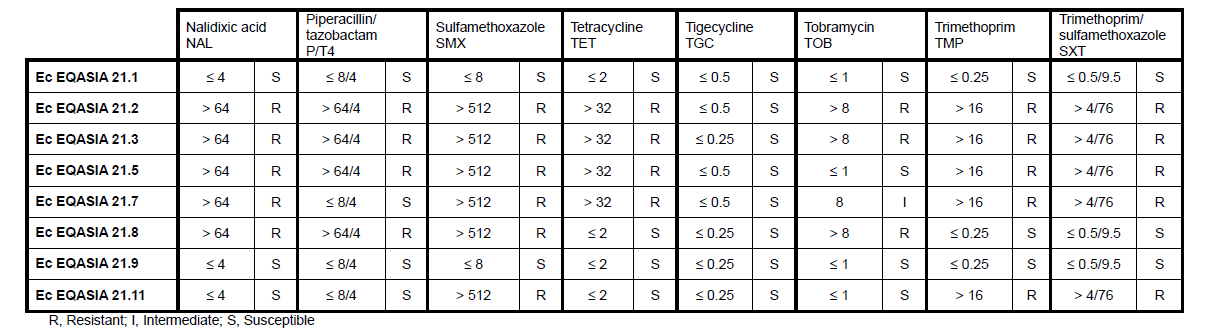
**

**
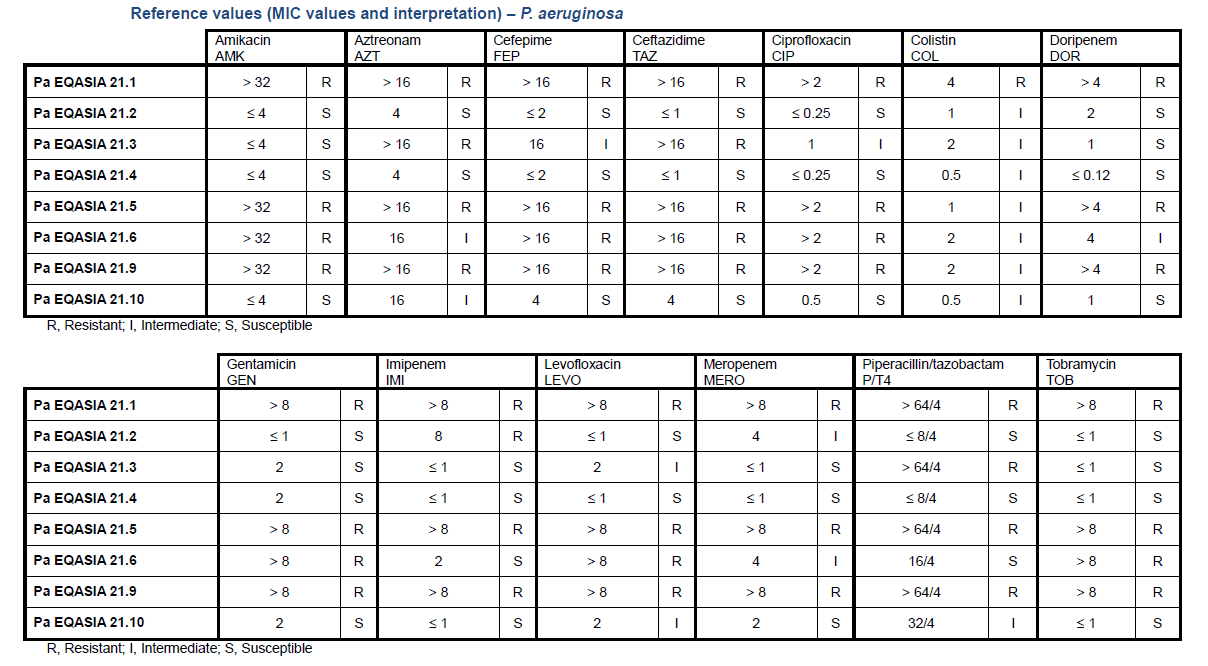
**

**
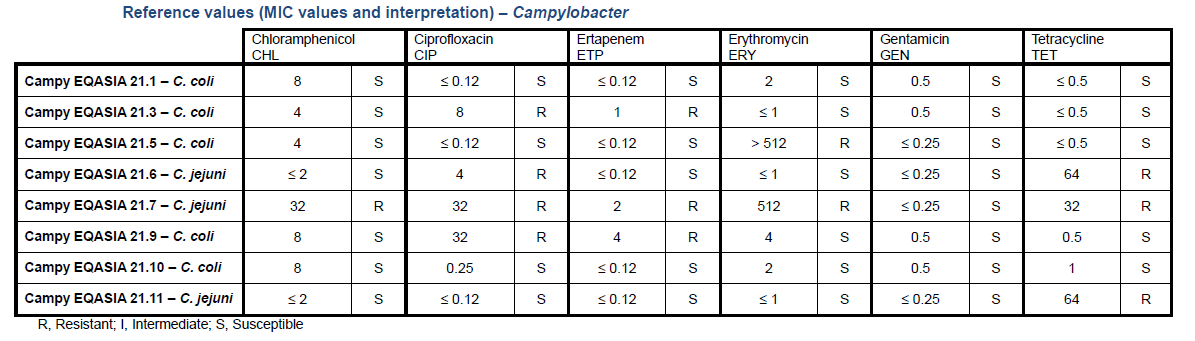
**

**
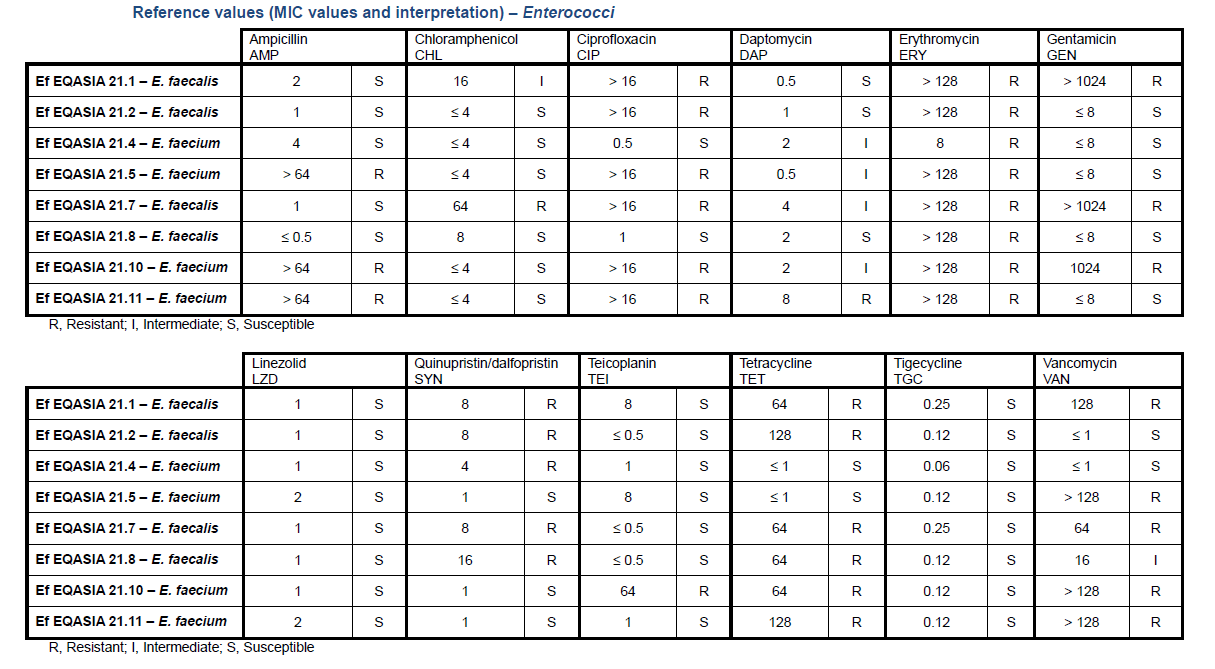
**

**
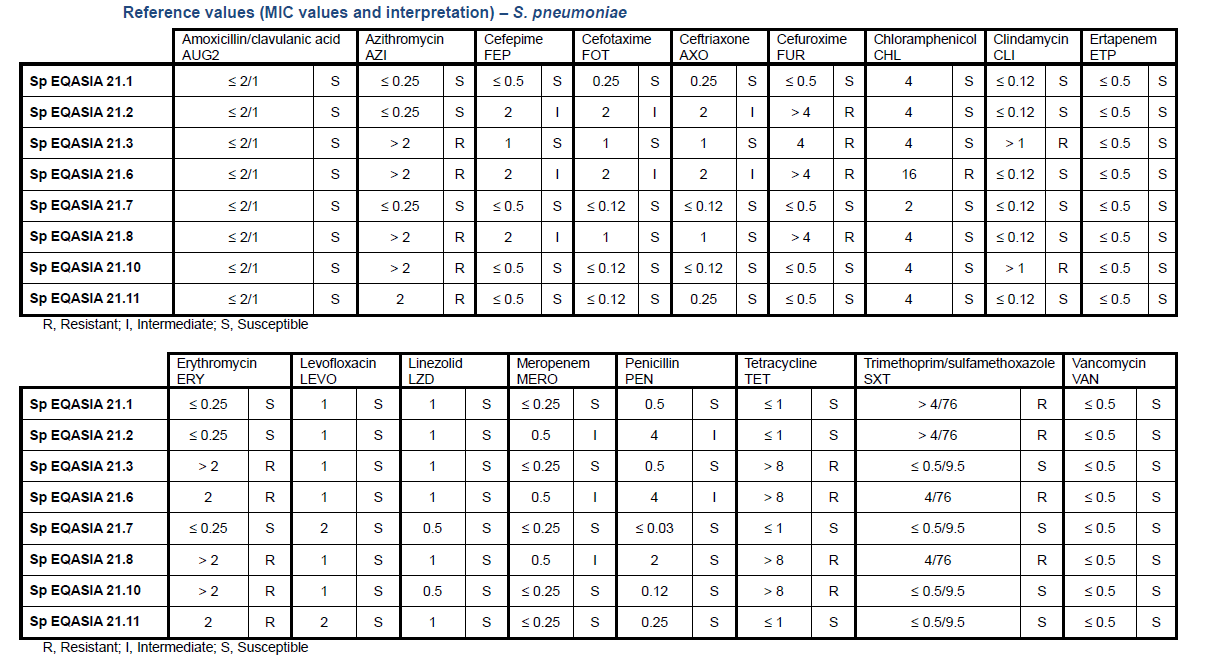
**

**EQASIA EQA4**

**
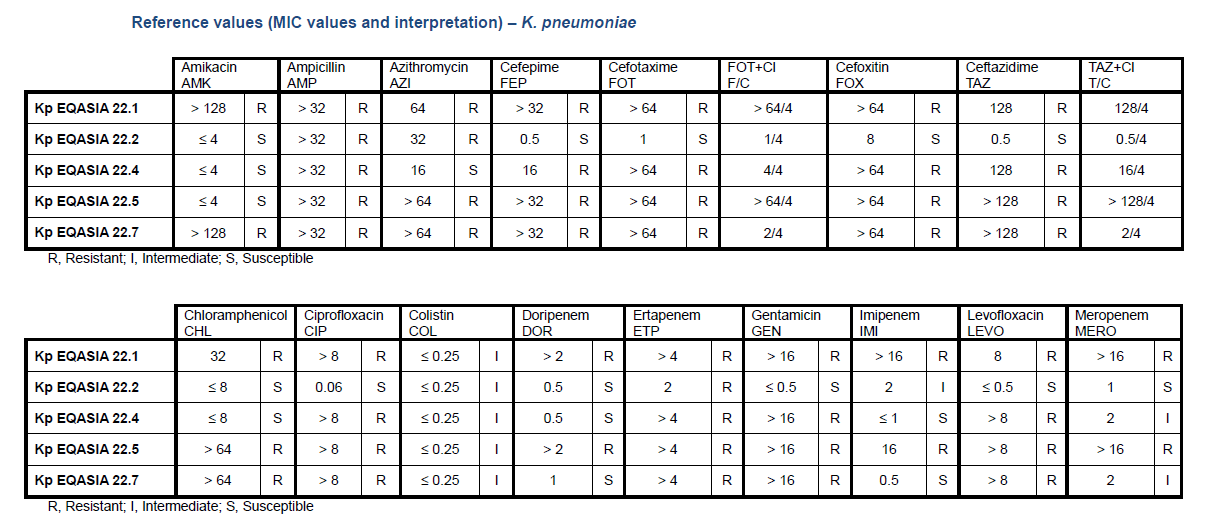
**

**
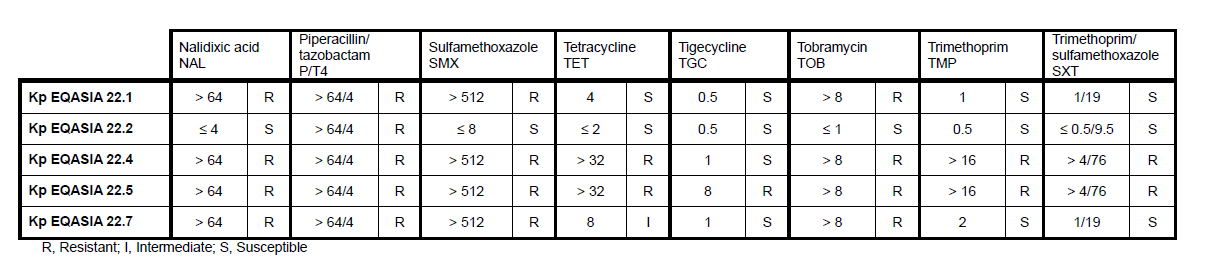
**

**
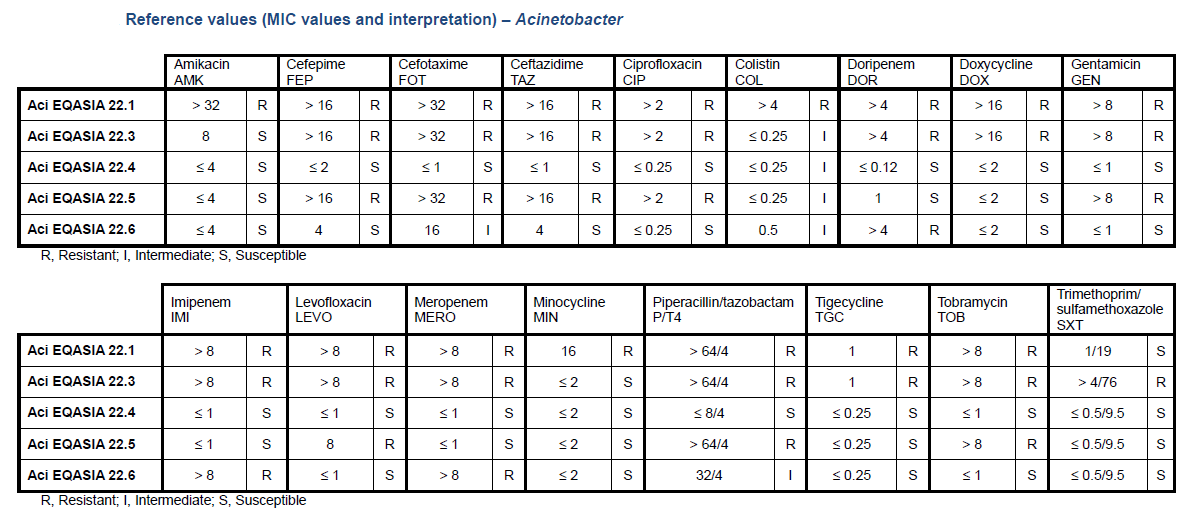
**

**
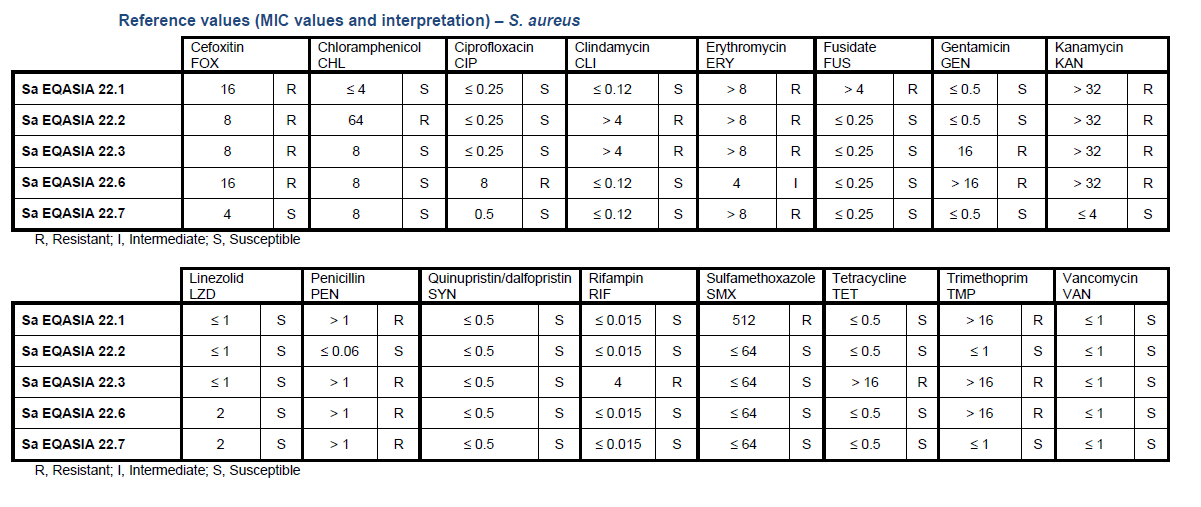
**

**EQASIA EQA5**

**
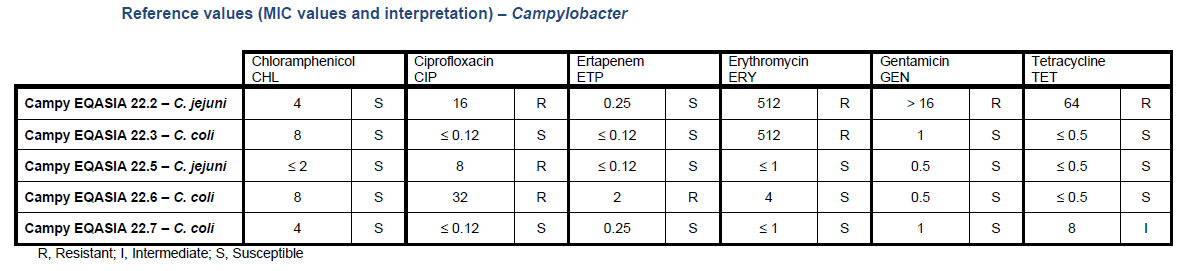
**

**
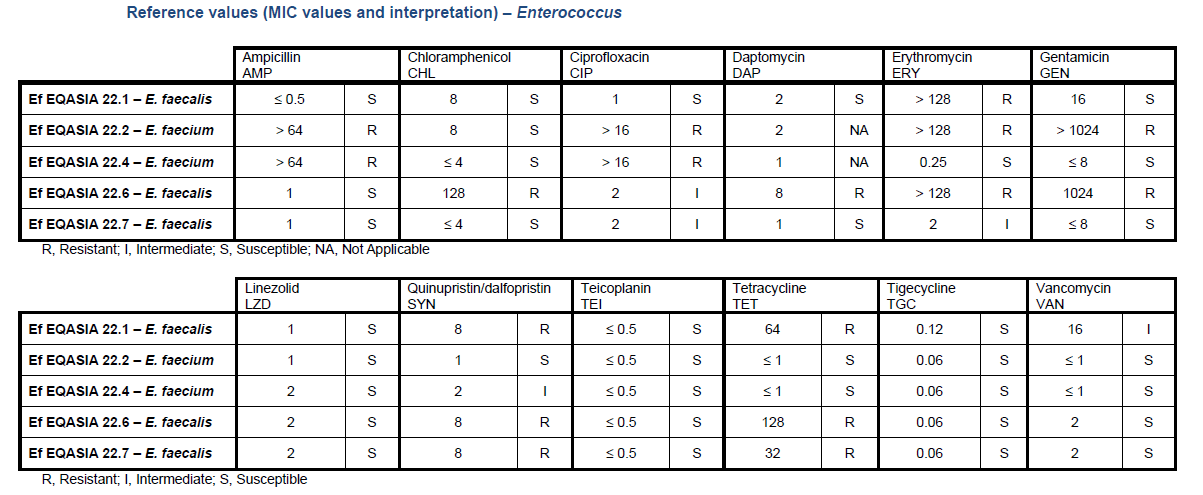
**

**
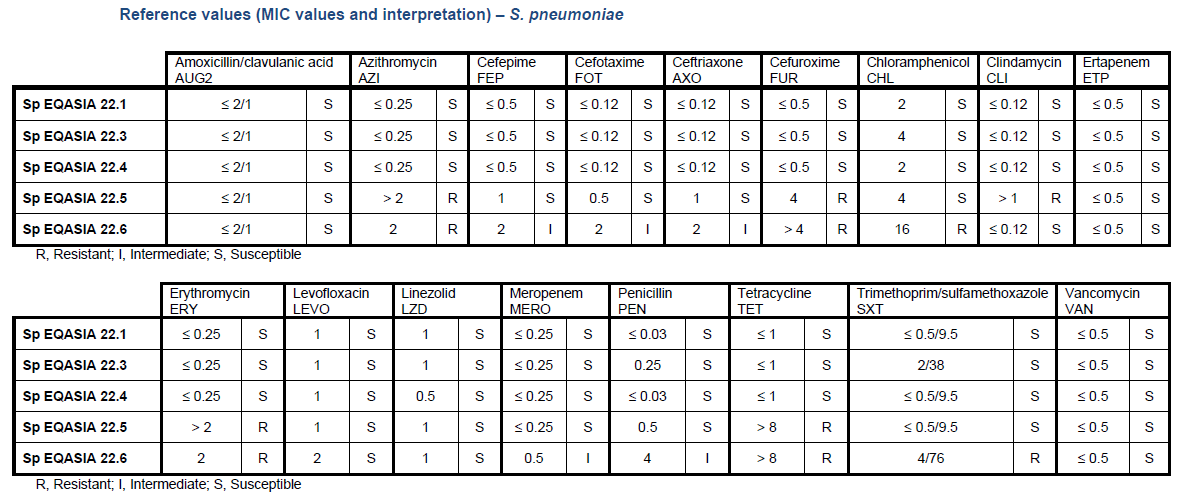
**

**EQASIA EQA6**

**
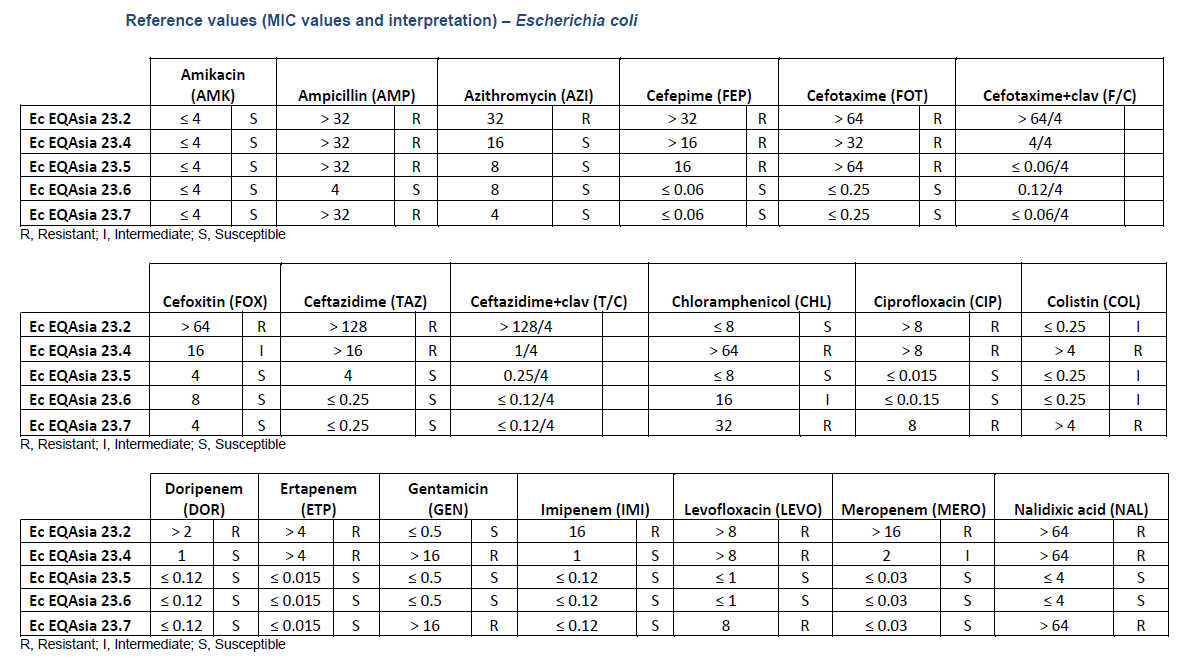
**

**
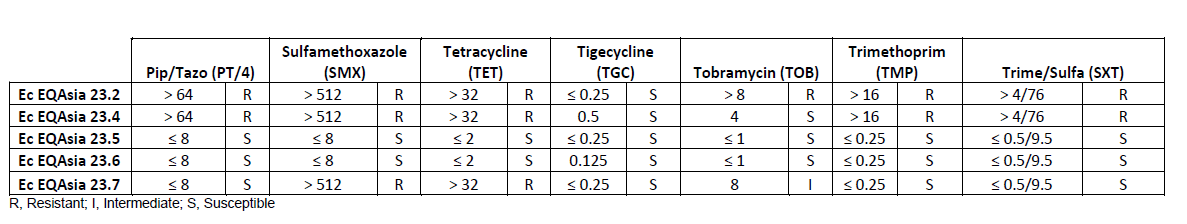
**

**
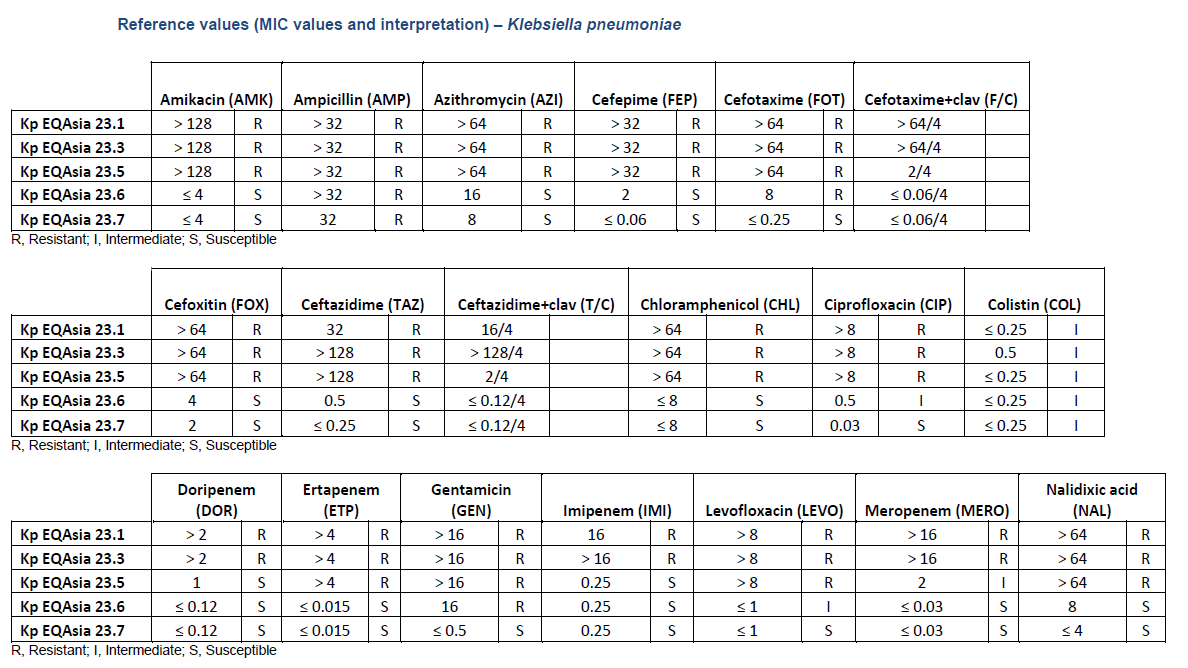
**

**
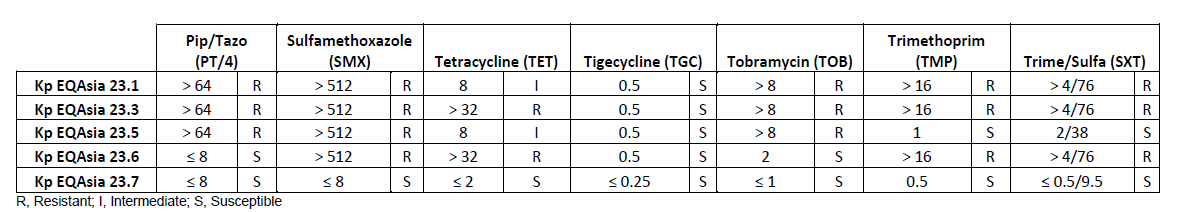
**

**
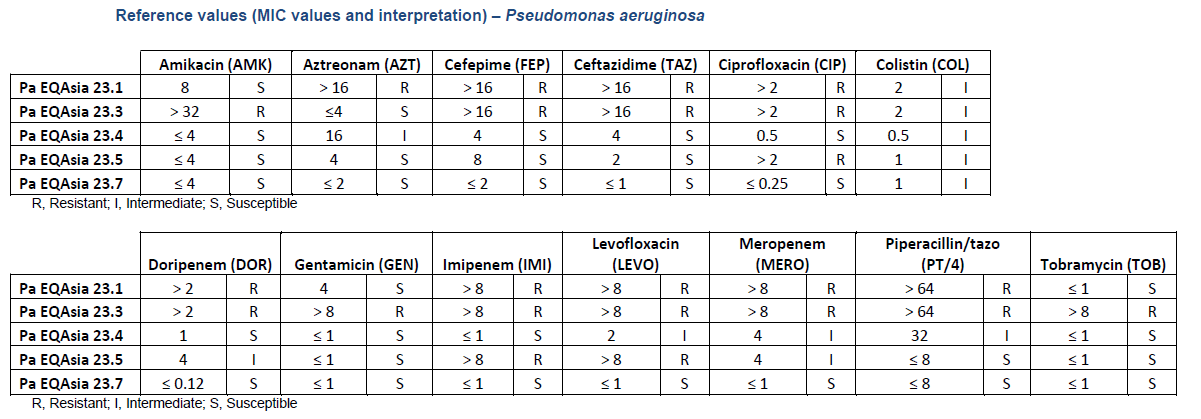
**

**
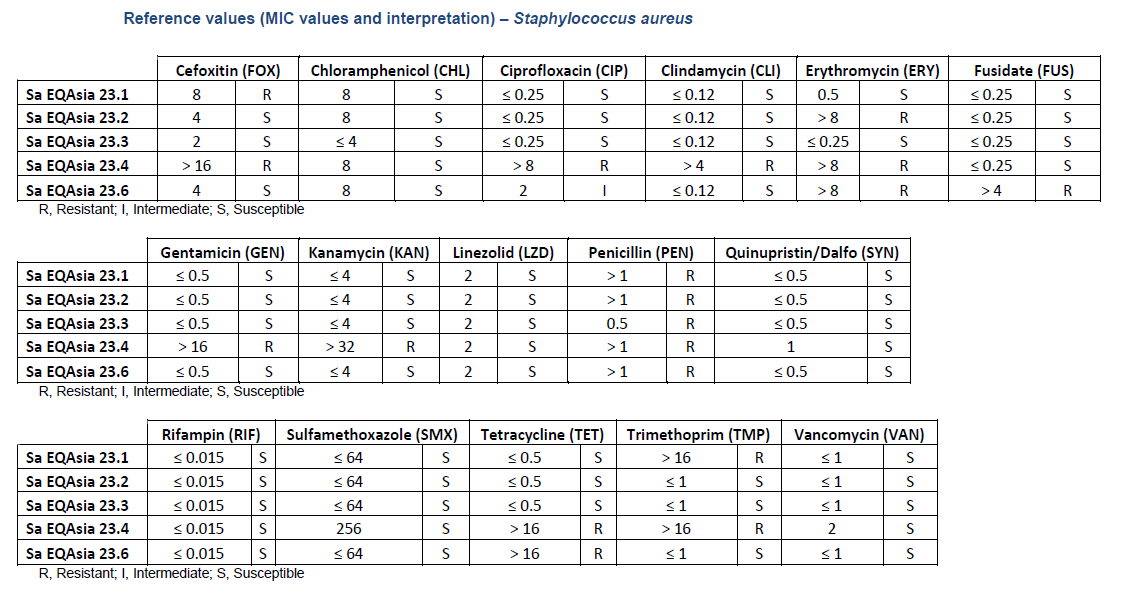
**


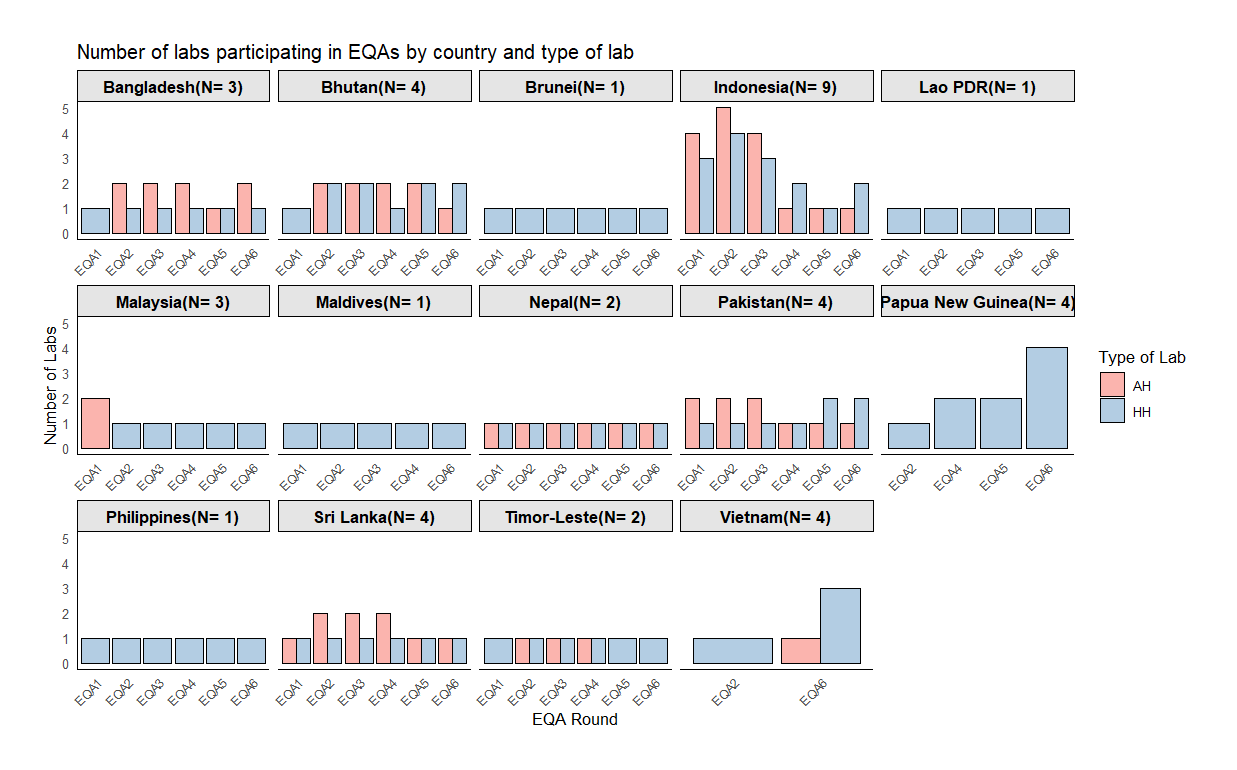


**Supplementary Figure 1.** Number of laboratories participating in EQASIA by country and type of laboratory; AH - animal health; HH - human health.


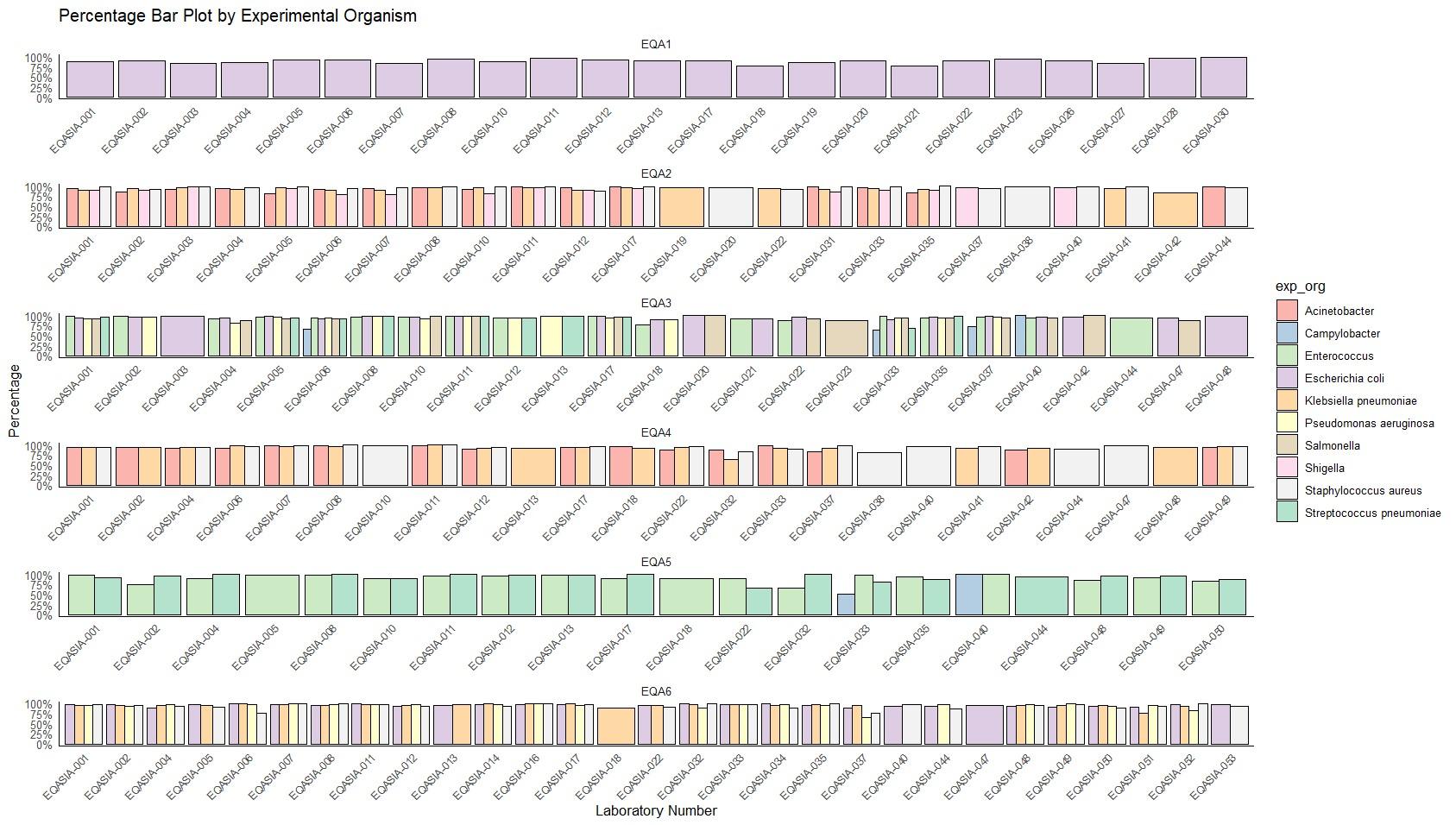


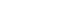


**Supplementary Figure 2.**  Overview of laboratories’ performance by EQA and pathogen.

**
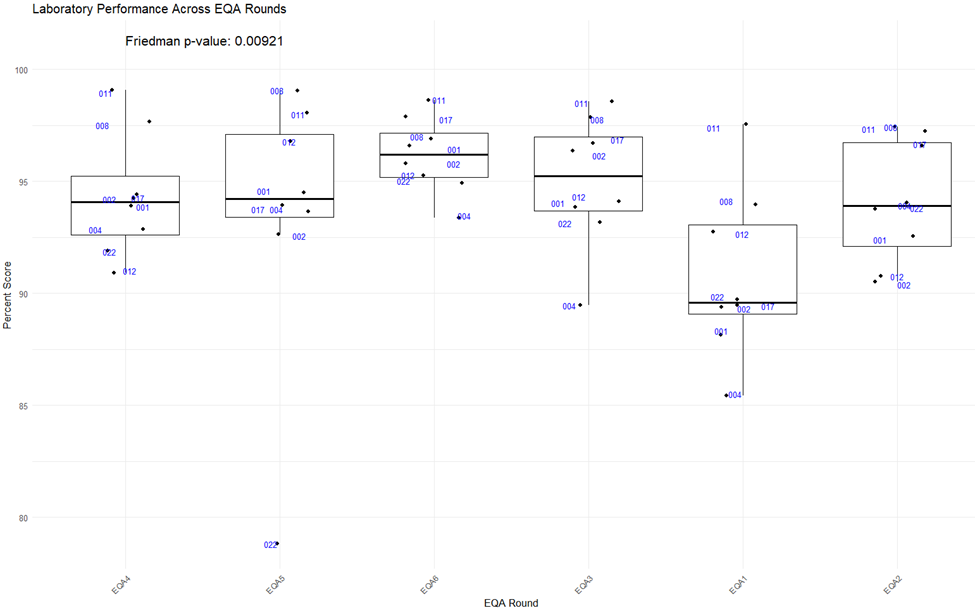
**

**Supplementary Figure 3.** The boxplot visualizes laboratory AST performance across six EQA rounds. Each box represents the interquartile range (IQR) of scores for each EQA round and the line the median (50^th^ percentile).
